# Hypercapnic respiratory failure during pregnancy due to polymyositis-related respiratory muscle weakness: a case report

**DOI:** 10.1186/s13256-017-1368-2

**Published:** 2017-07-26

**Authors:** Husain Shabbir Ali, Ibrahim Fawzy Hassan, Saibu George, Abdalrazig Elsadig Fadlelmula

**Affiliations:** Department of Medical ICU, Hamad General Hospital, P.O. Box 3050, Doha, Qatar

**Keywords:** Polymyositis, Inflammatory myopathy, Respiratory failure, Pregnancy

## Abstract

**Background:**

Polymyositis is a rare medical disorder complicating pregnancy. Ventilatory muscle weakness leading to respiratory failure is an uncommon manifestation of this autoimmune disease. We report a case of life-threatening hypercapnic respiratory failure due to polymyositis-related respiratory muscle weakness in a pregnant woman.

**Case presentation:**

A 31-year-old, African woman in her second trimester of pregnancy presented to the emergency department with fever, shortness of breath and muscle weakness. Initial investigations excluded pulmonary infection, thromboembolism, and cardiac dysfunction as the underlying cause of her symptoms. She developed deterioration in her level of consciousness due to carbon dioxide narcosis requiring invasive mechanical ventilation. Further workup revealed markedly elevated serum creatine kinase, abnormal electromyography and edema of her thigh muscles on magnetic resonance imaging. Diagnosis of polymyositis was confirmed by muscle biopsy. After receiving pulse steroid, intravenous immunoglobulins, and maintenance immunosuppressive therapy, our patient’s respiratory muscle function improved and she was weaned off mechanical ventilation. Despite good maternal recovery from critical illness, the fetus developed intrauterine growth retardation and distress necessitating emergency cesarian section.

**Conclusions:**

New-onset polymyositis during pregnancy presenting with respiratory failure is rare. Early diagnosis and prompt initiation of therapy is necessary to improve fetal and maternal outcomes.

## Background

Idiopathic inflammatory myopathies (IIM) are systemic connective tissue diseases which are characterized by symmetrical, proximal muscle weakness, decreased muscle endurance and chronic inflammation in muscle tissue [1]. Based on clinical and immunopathological features they are classified into dermatomyositis (DM), polymyositis (PM), immune-mediated necrotizing myopathy and sporadic inclusion body myositis [2]. The incidence of inflammatory myopathies as a whole ranges from 1.16 to 19/million/year and their prevalence ranges from 2.4 to 33.8 per 100,000 inhabitants [3].

Currently, there are few studies that describe pregnancy in DM/PM patients, and they are largely limited to case reports or studies with small samples. Thus, little is known about the effects of pregnancy on DM/PM, whether these patients find it harder to conceive or if pregnancy outcomes are adversely affected by myositis [4]. Respiratory failure due to respiratory muscle weakness is a rare complication of polymyositis, the prevalence of which is unknown [5]. We report a case of a patient with severe respiratory failure during pregnancy, due to alveolar hypoventilation resulting from polymyositis-related respiratory muscle weakness.

## Case presentation

**Fig. 1:**
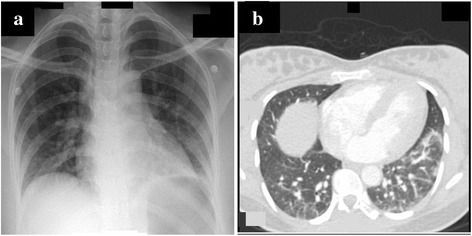
Chest imaging of the patient. **a** Chest X-ray on admission: prominent bronchovascular markings bilaterally. Hazy opacity noted in left lower lung field. **b** Chest computed tomography scan: bilateral basilar dependent atelectasis, more on the left side

A 31-year-old, African woman with no significant medical background presented to the emergency department with 6 weeks history of fever, difficulty in breathing, and weakness of proximal limb muscles. There was no history of skin rash. She was 18 weeks primigravida with an uneventful antenatal course. Initial assessment showed an averagely built female with a body mass index (BMI) of 23.7 kg/m^2^ (normal range: 18.5–24.9 kg/m^2^), temperature of 37.1 °C, heart rate of 90 beats/minute (regular), blood pressure of 144/84 mm Hg, respiratory rate of 22 breaths/minute and pulse oximetry 99% on room air. A respiratory system examination was unremarkable apart from reduced air entry at both lung bases. A neurological examination revealed intact higher mental functions, reduced power in both upper and lower limbs (proximal muscles 3/5 and distal muscles 4/5) [6], intact reflexes, sensations, cranial nerves, and cerebellar function. An abdominal examination showed gravid uterus appropriate for gestational age and a cardiovascular examination was unremarkable. Blood investigations revealed elevated alanine aminotransferase (ALT), aspartate aminotransferase (AST), creatine kinase (CK), troponin T (Table 1) and arterial blood gas on room air was suggestive of acute on chronic respiratory acidosis [pH – 7.31, partial pressure of carbon dioxide in arterial blood (PaCO_2_) – 58 mm Hg, partial pressure of oxygen in arterial blood (PaO_2_) – 79 mm Hg, bicarbonate (HCO_3_) – 28 mEq/L]. Our patient had normal blood leukocyte count and no organism was isolated from sputum, urine and blood cultures, excluding underlying infection. An electrocardiogram (ECG) and bedside 2D transthoracic echocardiography were not suggestive of acute coronary syndrome or structure heart lesions. A chest X-ray showed hazy opacity in her left lower lung field (Fig. 1a). A computed tomography (CT) scan of her chest revealed bilateral basilar dependent atelectasis and excluded pulmonary embolism, pleurisy, pneumothorax, consolidation, and interstitial lung disease (ILD) (Fig. 1b). A pelvic ultrasound showed a single viable fetus with size and weight appropriate for gestational age, fetal movements were seen, fetal cardiac pulsations recorded, and amniotic fluid was adequate. Our patient was admitted to the medical intensive care unit (MICU) for further investigation and management. She was started on intermittent noninvasive bilevel positive airway pressure (BiPAP), with inspiratory positive airway pressure (IPAP) of 10 cm H_2_O and expiratory positive airway pressure (EPAP) of 5 cm H_2_O, to improve her hypercapnic respiratory failure. However, our patient was not compliant to the prescribed noninvasive ventilation (NIV) therapy.

**Table 1:** Serum muscle enzyme levels of the patient

	On admission	Peak levels	On discharge	Normal range
ALT (U/L)	172	211	112	0–55
AST (U/L)	319	319	116	5–34
Troponin T (ng/L)	860	1557	NM	0–14
Creatine kinase (U/L)	1958	2338	680	29–168

*ALT* alanine aminotransferase, *U/L* units/liter, *AST* aspartate aminotransferase, *ng/L* nanogram/liter, *NM* not measured

**Fig. 2:**
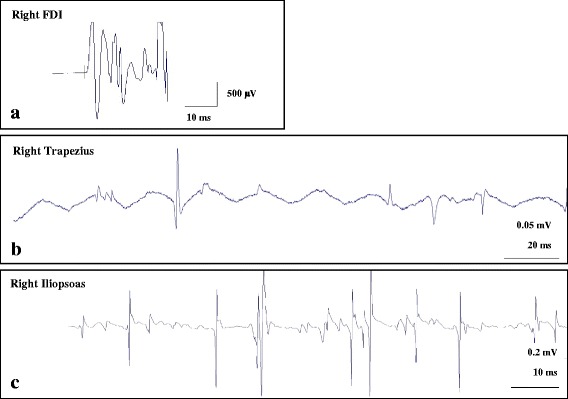
Needle electromyography. **a** Increased insertional activity of right first dorsal interosseous (FDI) muscle. **b** Spontaneous fibrillation potential and positive sharp waves of right trapezius muscle. **c** Myopathic motor unit potential and early recruitment of right iliopsoas muscle. *μV* microvolt, *ms* millisecond, *mV* millivolt

On the second day of hospitalization, our patient developed severe respiratory acidosis (pH: 7.13 and PaCO_2_: 101 mm Hg) leading to deterioration in her level of consciousness with Glasgow Coma Scale (GCS) score [7] of 8/15 (eye opening - to pain 2/4; verbal response - incomprehensible sounds 2/5; best motor response - withdraws from pain 4/6). She was initiated on invasive mechanical ventilation for severe hypercapnic respiratory failure. After correction of respiratory acidosis by invasive ventilation, our patient regained her level of consciousness. Bedside needle electromyography (EMG) revealed electrophysiological evidence of diffuse irritable myopathy in sampled muscles (Fig. 2). Based on Bohan and Peter criteria (Table 2) [8] patient was diagnosed as probable polymyositis and started on pulse steroid therapy with intravenous methylprednisolone 500 mg daily for 3 days, followed by maintenance oral prednisolone 60 mg daily and azathioprine 50 mg daily. After 5 days of initiating steroid therapy there was significant improvement in respiratory muscle function as evident from adequate gas exchange on low-setting pressure support ventilation [5 cm of water (H_2_O)] and increasing maximal inspiratory and expiratory pressures (MIP and MEP), so the patient was weaned off invasive ventilation. Extensive immunological workup did not reveal any positive autoantibodies. Four days postextubation there was deterioration in respiratory function (blood gas on room air: pH 7.25 and PaCO_2_ 93 mm Hg) necessitating initiation of intermittent noninvasive BiPAP (IPAP - 12 cm H_2_O and EPAP – 6 cm H_2_O), averaging 16 hours per day. After counselling the patient for compliance to NIV therapy, good adherence to BiPAP was maintained. Due to relapse in respiratory muscle weakness, intravenous immunoglobulins (IVIG) (0.4 grams/kg/day) were started for a total of five doses. After completion of immunoglobulin therapy, our patient was gradually weaned off NIV and transferred to the general medical ward.

**Table 2:** Bohan and Peter criteria for the diagnosis of polymyositis (PM) and dermatomyositis (DM)

1. Proximal muscle weakness, usually symmetrical
2. Elevated serum muscle enzymes
3. Electromyographic abnormalities
a. Common: myopathic potential – low amplitude, short duration and polyphasic action potentials
b. Characteristic triad: (i) myopathic potentials; (ii) fibrillations, positive sharp waves, increased insertional activity; (iii) complex repetitive discharges
4. Muscle biopsy findings typical of PM or DM: necrosis, phagocytosis, regeneration, inflammation
5. Dermatological features of DM: Gottron’s sign or papules, or heliotrope rash
Definite: PM – four criteria without rash. DM – four criteria including rash
Probable disease: PM – three criteria without rash. DM – three criteria including rash
Possible disease: PM – two criteria without rash. DM – two criteria including rash

**Fig. 3:**
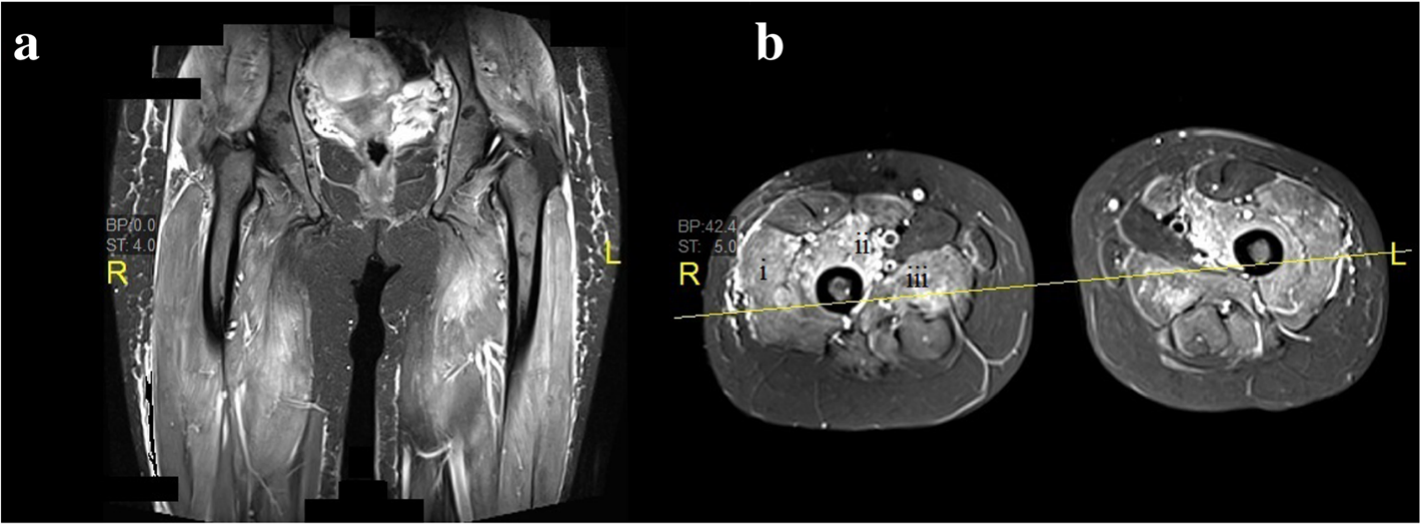
Magnetic resonance images of both thighs. **a** Coronal short T1 inversion recovery image. **b** Axial short T1 inversion recovery image. Diffuse hyperintensity in thigh muscles suggestive of edema – (i) vastus lateralis, (ii) vastus medialis, and (iii) adductor magnus

During her stay in the medical ward, our patient underwent magnetic resonance imaging (MRI) of both thighs, which showed edema of bilateral vastus lateralis, vastus medialis, adductor magnus, and gluteal muscles. (Fig. 3). A muscle biopsy taken from her right deltoid was suggestive of inflammatory myopathy (scattered fibers with necrosis and regeneration, macrophages invading necrotic planes, collection of mononuclear cells at perivascular sites in perimysium and endomysium, and increase in perimysial fibrous and fatty connective tissue). After the muscle histopathology report, our patient was confirmed to have definite PM (Table 2). Due to a rise in transaminases, not attributed to disease activity, azathioprine had to be stopped and our patient was kept on a tapering dose of oral corticosteroids. After 1 month of total hospitalization, our patient was discharged in good general condition with appropriate fetal growth, on maintenance oral prednisolone 20 mg daily and advised to follow up with her rheumatologist, obstetrician, and neurologist.

At 27 weeks of gestation, our patient was found to have intrauterine growth retardation (IUGR) on routine antenatal pelvic ultrasound. Fetal growth was estimated to be 24 weeks based on femoral length, biparietal diameter, head circumference, and fetal weight was calculated to be 627 grams. During a multidisciplinary meeting between obstetrician, neonatologist, rheumatologist, and anesthetist/intensivist, it was decided to continue the pregnancy with close monitoring of our patient in the obstetric ward. In case of clinical deterioration, an urgent delivery would be carried out using spinal anesthesia with administration of a stress dose of steroids. At 31 weeks gestation, cardiotocography (CTG) was nonreassuring with repeated unprovoked decelerations and our patient underwent emergent lower segment cesarean section (LSCS) using combined spinal and epidural anesthesia. A baby boy with severe IUGR, weighing 1160 grams was delivered and immediately required invasive ventilation for respiratory distress syndrome (RDS). The baby had a prolonged neonatal intensive care unit (NICU) stay complicated by Acinetobacter sepsis. He was gradually weaned of mechanical ventilation and transferred to the neonatal ward. The mother had an uneventful postoperative course and was discharged after 7 days of delivery with follow-up in the obstetric and rheumatology clinics.

## Discussion

Acute respiratory failure occurs in less than 0.1% of pregnancies but the potential maternal and fetal consequences can be devastating [9]. The gravid woman undergoes a number of respiratory adaptations, some of which increase her risk for respiratory compromise. Progesterone stimulates a 30% increase in minute ventilation, which is achieved by an increase in tidal volume without significant change in the respiratory rate. Maternal PaCO_2_ drops from a range of about 36–44 mm Hg to a range of 28–32 mm Hg, but renal compensation helps maintain arterial pH between 7.40 and 7.47 [10]. Thus, a seemingly minor increase in PaCO_2_ may reflect significant respiratory compromise in a pregnant woman.

Lungs are the most common extramuscular organs affected in polymyositis-dermatomyositis (40% of patients) and their involvement is associated with significant morbidity and mortality [11]. Pulmonary complications include ILD, aspiration, infectious pneumonia, drug-induced lung disease, diffuse alveolar hemorrhage, pneumothorax, pulmonary arterial hypertension, and ventilatory muscle weakness [5]. A few cases of polymyositis-induced ventilatory muscle weakness leading to acute respiratory failure in nonpregnant women have been previously reported [12]. Table 3 summarizes the two previous case reports of respiratory failure due to inflammatory myopathy-related respiratory muscle weakness during pregnancy and allows comparison with our patient [13, 14].

**Table 3:** Cases of inflammatory myopathy-related ventilatory muscle weakness leading to acute respiratory failure during pregnancy

Reference	Age of the patient	Type of inflammatory myopathy	Overlapping connective tissue diseases	Gestational age at presentation	Onset of respiratory failure	Immunosuppressive treatment	Maternal outcome
Ishikawa *et al*. [13]	33 years	Polymyositis	No	31 weeks	Immediately post LSCS	Corticosteroids	Good recovery
Nozaki *et al.* [14]	31 years	Dermatomyositis	No	18 weeks	During LSCS	Corticosteroids and IVIG	Good recovery
Our patient	31 years	Polymyositis	No	18 weeks	18 weeks	Corticosteroids, IVIG and AZA	Good recovery

*LSCS* lower segment cesarean section, *IVIG* intravenous immunoglobulins, *AZA* azathioprine

The use of NIV in patients with neuromuscular diseases has been increasing over the past decade. Weakness can affect three main respiratory muscle groups: inspiratory muscles (diaphragm, parasternal, scalene, and accessory muscles); expiratory muscles (external intercostal and abdominal muscles); and muscles that innervate the upper airways (palatine, pharyngeal, and genioglossal muscles). The mechanisms by which NIV produces beneficial effects are not fully understood, although the following hypothesis have been proposed: (a) respiratory muscle rest, (b) improved central respiratory response to carbon dioxide (CO_2_), (c) changes in pulmonary mechanics, and (d) improved sleep architecture [15].

The mainstay of therapy for IIM is immunosuppression, physical therapy, and avoidance of complications. First-line pharmacological therapy is corticosteroids, generally starting with prednisolone at 1 mg/kg/day, with eventual taper after several months to the lowest dose to maintain a remission. In patients with severe disease, methylprednisolone 1 gram/day is given intravenously for 3–5 days at onset. Second-line treatments can be added to the therapeutic regimen several months after the start of prednisolone, or in severe disease, begun immediately. Data are limited regarding what agent to use but choices include azathioprine, methotrexate, IVIG, mycophenolate mofetil, cyclophosphamide, immunophilin inhibitors, and rituximab [16]. The safety of treatment with immunosuppressive drugs during pregnancy is a major concern for both patients and their providers. The potential for fetotoxic effects of immunosuppressive medications that are commonly used to treat systemic autoimmune diseases must be weighed against the need for control of disease activity during pregnancy and the postpartum period, since active disease can be an independent risk factor for adverse pregnancy outcomes [17]. Cooper and colleagues have reported no evidence of a large increase in risk of adverse fetal outcomes from first trimester exposure to immunosuppressive medications, though confidence intervals for risk ratios were wide [18].

In gravid women with IIMs, fetal prognosis parallels activity of the maternal disease. In patients with pre-existing quiescent disease, little apparent risk to the mother or fetus is observed. This is in contrast to new onset of disease during pregnancy or exacerbation during pregnancy, for which a significantly worse outcome is noted [19]. In our case, despite good recovery of maternal respiratory function and muscle power, the fetus developed IUGR and had a complicated hospital course. Our adverse fetal outcome could have been multifactorial: active disease during pregnancy, derangements in maternal gas exchange and acid-base balance, or due to the effect of immunosuppressive agents.

## Conclusions

Hypercapnic respiratory failure due to alveolar hypoventilation as a sequelae of ventilatory muscle weakness is an unusual manifestation of IIMs. Given the low incidence of polymyositis, its implications for pregnancy are poorly understood. For new-onset disease during pregnancy, prompt diagnosis and initiation of therapy can improve maternal and fetal outcomes. Corticosteroids are the mainstay of treatment and second-line immunosuppressive drugs can be considered after assessing the risks/benefits. A multidisciplinary team approach involving rheumatologist, neurologist, obstetrician, neonatologist, and intensivist/anesthetist should be the standard of care in such rare and challenging cases.

## Abbreviations

μV: Microvolt
ALT: Alanine aminotransferase
AST: Aspartate aminotransferase
BiPAP: Bilevel positive airway pressure
BMI: Body mass index
CK: Creatine kinase
cm: Centimeter
CO_2_: Carbon dioxide
CT: Computed tomography
CTG: Cardiotocography
DM: Dermatomyositis
ECG: Electrocardiogram
EMG: Electromyography
EPAP: Expiratory positive airway pressure
FDI: First dorsal interosseous
GCS: Glasgow Coma Scale
H_2_O: Water
HCO_3_: Bicarbonate
IIM: Idiopathic inflammatory myopathy
ILD: Interstitial lung disease
IPAP: Inspiratory positive airway pressure
IUGR: Intra-uterine growth retardation
IVIG: Intravenous immunoglobulins
LSCS: Lower segment caesarean section
MEP: Maximal expiratory pressure
MICU: Medical intensive care unit
MIP: Maximal inspiratory pressure
MRI: Magnetic resonance imaging
ms: Millisecond
mV: Millivolt
NICU: Neonatal intensive care unit
NIV: Non-invasive ventilation
PaCO_2_: Partial pressure of carbon dioxide in arterial blood
PaO_2_: Partial pressure of oxygen in arterial blood
PM: Polymyositis
RDS: Respiratory distress syndrome
